# Supplementary material for: Determinants of maternal near miss among women admitted to maternity wards of tertiary hospitals in Southern Ethiopia, 2020: A hospital-based case-control study
Source: PLoS One. 2021 May 17;16(5):e0251826. doi: 10.1371/journal.pone.0251826 (PMC8128231; doi:10.1371/journal.pone.0251826)
Supplement: S1 Data — (DOCX) [file pone.0251826.s001.docx]

## Annex2. English Version of Questionnaire

| **S No** | | **Questions** | | | | **Response** | | | | | **Skip** |
| --- | --- | --- | --- | --- | --- | --- | --- | --- | --- | --- | --- |
| **Part I: Identifications** | | | | | | | | | | |  |
| 101 | | Hospital Name | | | | | 1. Butajira tertiary hospital 2. Wolita Soddo tertiary hospital 3. Wolkite tertiary hospital 4. Worabe tertiary hospital 5. Nigist Eleni Mohammed Memorial tertiary hospital | | | |  |
| 102 | | Hospital code | | | | | **____/___/____** | | | |  |
| 103 | | Date form filled to start | | | | | ____/___/____ | | | |  |
| 104 | | Woreda /District | | | | | ________________________ | | | |  |
| 105 | | Kebele | | | | | ________________________ | | | |  |
| 106 | | Admission date | | | | | _____/____/____EC | | | |  |
| 107 | | Maternity record number/Medical Reg. Number | | | | | _________________ | | | |  |
| **Part II Socio demographic and economic characteristics of respondents** | | | | | | | | | | | **Skip** |
| 201 | Where is your place of residence | | | 1. Urban 2. Rural | | | | | | |  |
| 202 | How old are you? | | | 1. [___________]  2. I don’t know------------ | | | | | | |  |
| 203 | What is your marital status? | | | 1. Married | | | | 1. Divorced | | |  |
|  |  |  |  | 1. Unmarried/single | | | | 1. Widowed | | |  |
| 204 | What is your religion? | | | 1. Orthodox | | | | 1. Protestant | | |  |
|  |  |  |  | 1. Catholic | | | | 1. Muslim | | |  |
|  |  |  |  | 1. Other | | | |  | | |  |
| 205 | What is your ethnicity? | | | 1. Hadiya | | | | 1. Kembata | | |  |
|  |  |  |  | 1. Siltei | | | | 1. Guraghe | | |  |
|  |  |  |  | 1. Wolaita | | | | 1. Others | | |  |
| 206 | What is your educational status? | | | 1. No formal education | | | | 1. Primary education (1-8^th^ | | |  |
|  |  |  |  | 1. 2ndary education (9-12^th^ ) | | | | 1. College and above | | |  |
| 207 | What is your occupation? | | | 1. House wife | | | | 1. Merchant | | |  |
|  |  |  |  | 1. Farmer | | | | 1. Daily laborer | | |  |
|  |  |  |  | 1. Government employer | | | | 1. Others | | |  |
| 208 | What is the educational level of your husband? | | | 1. No formal education | | | | 2. Primary education (1-8^th^ | | |  |
|  |  |  |  | 3. 2ndary education (9-12^th^ ) | | | | 4. College and above | | |  |
| 209 | What is his present Occupation? | | | 1. Farmer | | | | 1. Merchant | | |  |
|  |  |  |  | 1. Government employer | | | | 1. Daily laborer | | |  |
|  |  |  |  | 1. Others | | | | 1. No work | | |  |
| 210 | How many members are there within the family?(family size) | | | | | | | [________________] | | |  |
| 211 | **Wealth index measurement** | | | | | | | Yes= 1 | No=0 | |  |
| 1 | Does your household have: | | | 1. Electricity | | | |  |  | |  |
|  |  |  |  | 1. Radio | | | |  |  | |  |
|  |  |  |  | 1. Television | | | |  |  | |  |
|  |  |  |  | 1. mobile telephone | | | |  |  | |  |
|  |  |  |  | 1. table | | | |  |  | |  |
|  |  |  |  | 1. chair | | | |  |  | |  |
|  |  |  |  | 1. Bed | | | |  |  | |  |
| 2 | Does anyone of your household member have? | | | 1. Bicycle | | | |  |  | |  |
|  |  |  |  | 1. An animal-drawn cart | | | |  |  | |  |
|  |  |  |  | 1. Motorcycle | | | |  |  | |  |
|  |  |  |  | 1. Bajaj | | | |  |  | |  |
|  |  |  |  | 1. A car/truck? | | | |  |  | |  |
| 3 | Do you have private home? | | | | | | |  |  | |  |
| 4 | Main material of the roof (observe) | | | | | | | 1. Grass 2. Metal/corrugated iron | | |  |
| 5 | What is the main source of drinking water for your household? (circle or tick on the options) | | | | | | | 1. Water from spring/ river/ pond 2. Dug well 3. Pressure Piped 4. Tap water | | |  |
| 6 | What type of fuel does your household mainly use for Cooking?(code based on the respective number) | | | | | | | 1. Animal dung 2. Wood 3. Charcoal 4. Electricity | | |  |
| 7 | Does any member of this household have a bank or microfinance saving account | | | | | | | 1. No 2. Yes | | |  |
| 8 | How many of the following animals does the house hold have? (in number) | | | | | | | Milk Cows | |  |  |
|  |  |  |  |  |  |  |  | Ox | |  |  |
|  |  |  |  |  |  |  |  | Hen | |  |  |
|  |  |  |  |  |  |  |  | Goat/Sheep | |  |  |
|  |  |  |  |  |  |  |  | Donkey/Horse/Mule | |  |  |
| 9 | Does the household have cultivated agricultural land? | | | | | | | 1. No 2. Yes | | |  |
| 10 | If yes, in Hectares [__________________________] | | | | | | | | | |  |
| 11 | Did you rent/lease out land over the last 12 months? | | | | | | | 1. No 1. Yes | | |  |
| 12 | In the past 12 months how many quintals did you got? [list amount produced for each crop] | | | | | | | 1. Teff |  | |  |
|  |  |  |  |  |  |  |  | 2. Barely |  | |  |
|  |  |  |  |  |  |  |  | 3. Wheat |  | |  |
|  |  |  |  |  |  |  |  | 4. Maize |  | |  |
| **PARTIII: OBSTETRIC CHARACTERISTICS OF RESPONDENTS** | | | | | | | | | | |  |
|  | | | Age at marriage | |  | | | | | |  |
|  | | | Age at first pregnancy | |  | | | | | |  |
|  | | | Number of pregnancies (gravidity)? | |  | | | | | |  |
|  | | | Number of birth orders (parity)? | |  | | | | | |  |
|  | | | Gestational age at last delivery | | 1. <37 week 2. 37-41 week 3. ≥42 week | | | | | |  |
|  | | | The duration between the current birth and the preceding birth in months? | | **/_______________/** | | | | | |  |
|  | | | Planning status of your last pregnancy while you got pregnant for the last time? | | 1. I had a plan and desire to that pregnancy | | | | | |  |
|  |  |  |  |  | 1. The pregnancy occurred earlier than desired) | | | | | |  |
|  |  |  |  |  | 1. The pregnancy occurred when no or more children were desired | | | | | |  |
|  | | | Did you Have History of obstetric complication | | 1. Yes 2, No | | | | | | **401** |
|  | | | What are those complications | | 1.  2.  3.  4. | | | | | |  |
|  | | | Ever had abortion | | 1. Yes 2. No | | | | | |  |
|  | | | Induction of labor | | 1. Yes 2. No | | | | | |  |
|  | | | Birth outcome of last pregnancy | | 1. Live birth 2. Still birth | | | | | |  |
|  | | | Birth weight in gm. | |  | | | | | |  |
| **HEALTH SERVICE RELATED CHARACTERISTICS OF RESPONDENTS** | | | | | | | | | | | |
|  | | | Did you have ANC visit while you were pregnant? | | 1. Yes  2. No | | | | | | **308** |
|  | | | How many times you had got the visit? | | [______________] | | | | | |  |
|  | | | Timing of ANC booking | | 1. Early booking (≤12 weeks)  2. Late booking (>12 weeks) | | | | | |  |
|  | | | Where was place of your last ANC visit? | | 1. Health center | | | 2. hospital | | |  |
|  |  |  |  |  | 3. Health post | | | 4. other | | |  |
|  | | | Where did you give your last birth | | 1. Health center | | | 1. Hospital | | |  |
|  |  |  |  |  | 1. Health post | | | 1. Home | | |  |
|  | | | In what Mode of delivery you got your child? | | 1. SVD | | | 2. Instrumental deliver | | |  |
|  |  |  |  |  | 3.C/S | | |  | | |  |
|  | | | History of previous cesarean section | | 1. Yes 2. No | | | | | |  |
|  | | | Well birth preparedness and complication readiness | | 1. Yes 2. No | | | | | |  |
|  | | | In what circumstance she got well prepared (Yes=1, NO=0) | | Ascertained place for birth | | | | | |  |
|  |  |  |  |  | Identified birth attendants | | | | | |  |
|  |  |  |  |  | Set aside money | | | | | |  |
|  |  |  |  |  | Identified emergency transportation | | | | | |  |
|  |  |  |  |  | Identified labor and birth companion | | | | | |  |
|  |  |  |  |  | Identified nearby health facility | | | | | |  |
|  |  |  |  |  | Identified blood donors if required | | | | | |  |
|  |  |  |  |  | Identified care giver to children’s at home | | | | | |  |
|  | | | Do you know at least one danger sign related with pregnancy and child birth | | 1. Yes 2. No | | | | | |  |
|  | | | From where you were referred to this hospital | | 1. Health facility referred  2. self-referred from home | | | | | |  |
|  | | | Which means of transportation you used | | 1. Ambulance 2. Private vehicle  2. On foot 3. Others | | | | | |  |
|  | | | How long does it take to decide for seeking maternal health care | | [--------------------------]hours | | | | | |  |
|  | | | How long does it takes from your decision to reaching to health facility | | [--------------------------]hours | | | | | |  |
|  | | | How long does it take for receiving care after your arrival to this hospital | | [--------------------------]hours | | | | | |  |
|  | | | Who will decide when you want to go to health facility for maternity services? | | 1. Myself 2. Me and my husband   3. My husband | | | | | |  |
|  | | | Did you stayed at maternity waiting room? | | 1. Yes  2.No | | | | | | 506 |

**PART- Preexisting Chronic Medical Disorders**

| 401 | Did faced Hypertensive disorders of pregnancies? | 1. Yes 2. No | | 403 |
| --- | --- | --- | --- | --- |
| 402 | What type of HDPs( record review) | 1. Chronic HTN | 2. Pre-Eclampsia |  |
|  |  | 2. Eclampsia | 4. Gestational HTN |  |
| 403 | Did you sustained Gush of fluid before the onset of labor | 1. Yes 2. No | |  |
| 404 | Did you sustained Vaginal bleeding before delivery | 1. Yes 2. No | |  |
| 405 | Did you diagnosed with anemia during your pregnancy | 1. Yes 2. No | |  |
| 406 | Did you diagnosed with DM during your pregnancy | 1. Yes 2. No | |  |
|  | Did you diagnosed with syphilis during your last pregnancy | 1. Yes 2. No | |  |
| 408 | Do you have At least one preexisting medical problem | 1. Yes 2. No | |  |

## Annex3: Abstraction checklist for Neonatal near miss cases

| **Part VI: Maternal Near-Miss case abstraction checklist** | | | |
| --- | --- | --- | --- |
| 1 | **Severe complications / potentially life-threatening conditions** | Yes =1 | No=0 |
| 1.1 | Severe postpartum haemorrhage |  |  |
| 1.2 | Severe preeclampsia |  |  |
| 1.3 | Eclampsia |  |  |
| 1.4 | Sepsis or severe systemic infection |  |  |
| 1.5 | Ruptured uterus |  |  |
| 1.6 | Severe anemia |  |  |
| 2 | **Critical interventions or intensive care unit admission** |  |  |
| 2.1 | Use of blood products (includes any blood transfusion) |  |  |
| 2.2 | Interventional radiology (uterine artery embolization) |  |  |
| 2.3 | Laparotomy |  |  |
| 2.4 | Admission to Intensive Care Unit |  |  |
| **3** | **Organ dysfunction / life-threatening conditions** | | |
| 3.1 | Cardiovascular dysfunction (shock, sudden absence of pulse and loss of consciousness, use of continuous vasoactive drugs) |  |  |
| 3.2 | Respiratory dysfunction(acute cyanosis, severe tachypnea (respiratory rate>40 bpm), severe bradypnea (respiratory rate<6 bpm), or intubation and ventilation not related to anaesthesia) |  |  |
| 3.3 | Renal dysfunction(oliguria non responsive to fluids or diuretics, or severe acute azotemia (creatinine ­3.5mg/dL) |  |  |
| 3.4 | Hepatic dysfunction (jaundice, or severe acute hyperbilirubinemia (bilirubin >6.0mg/dL) |  |  |
| 3.5 | Neurological dysfunction (prolonged unconsciousness / coma (lasting >12 hours) |  |  |
| 3.6 | Uterine dysfunction (haemorrhage or infection leading to hysterectomy) |  |  |
| 3.7 | Coagulation/hematological dysfunction (failure to form clots, or massive transfusion of blood or red cells (­ 5 units) or severe acute thrombocytopenia (<50,000 platelets/ml) |  |  |
| **Is the newborn considered near-miss?** | |  |  |

**THANK YOU!!!!!**
