# Supplementary material for: Determinants of maternal near miss among women admitted to maternity wards of tertiary hospitals in Southern Ethiopia, 2020: A hospital-based case-control study
Source: PLoS One. 2021 May 17;16(5):e0251826. doi: 10.1371/journal.pone.0251826 (PMC8128231; doi:10.1371/journal.pone.0251826)
Supplement: Attachment — Submitted filename: Response to Reviewers.docx [file pone.0251826.s003.docx]

**AUTHORS’ RESPONSE TO ACADEMIC EDITOR AND REVIEWERS**

1. **AUTHOR’S RESPONSE TO EDITOR-Dr. SAMSON GEBIREMEDIHIN**

**Dear: Ricardo Q. Gurgel, PhD(Academic Editor )**

We thank you for a thorough reading and constructive comments and suggestions of our manuscript and for the opportunity to revise and resubmit. We are pleased to submit the revised research article “**Determinants of Maternal Near Miss among Women Admitted to Maternity Wards of Tertiary Hospitals in Southern Ethiopia, 2020: A Hospital-Based Case-control Study**” for your consideration in the special collection of Plos One. On the following pages, you will find our response to the editor’s comments. On behalf of my co-authors, I thank you for your consideration of this resubmission. We appreciate your time and look forward to your response.

Sincerely,

Aklilu Habte(MPH)(corresponding author)

[**aklilihabte57@gmail.com**](mailto:aklilihabte57@gmail.com)

**AUTHORS’ RESPONSE TO ACADEMIC EDITOR**

PONE-D-20-40965

Determinants of Maternal Near Miss among Women Admitted to Maternity Wards of Tertiary Hospital in Southern Ethiopia, 2020: A Hospital-Based Case-control Study

PLOS ONE

Dear Dr. Hailegebireal,

Thank you for submitting your manuscript to PLOS ONE. After careful consideration, we feel that it has merit but does not fully meet PLOS ONE’s publication criteria as it currently stands. Therefore, we invite you to submit a revised version of the manuscript that addresses the points raised during the review process.

Please submit your revised manuscript by Apr 22 2021 11:59PM. If you will need more time than this to complete your revisions, please reply to this message or contact the journal office at plosone@plos.org. When you're ready to submit your revision, log on to https://www.editorialmanager.com/pone/ and select the 'Submissions Needing Revision' folder to locate your manuscript file.

Please include the following items when submitting your revised manuscript:

•A rebuttal letter that responds to each point raised by the academic editor and reviewer(s). You should upload this letter as a separate file labeled 'Response to Reviewers'.

•A marked-up copy of your manuscript that highlights changes made to the original version. You should upload this as a separate file labeled 'Revised Manuscript with Track Changes'.

•An unmarked version of your revised paper without tracked changes. You should upload this as a separate file labeled 'Manuscript'.

If you would like to make changes to your financial disclosure, please include your updated statement in your cover letter. Guidelines for resubmitting your figure files are available below the reviewer comments at the end of this letter.

If applicable, we recommend that you deposit your laboratory protocols in protocols.io to enhance the reproducibility of your results. Protocols.io assigns your protocol its own identifier (DOI) so that it can be cited independently in the future. For instructions see: http://journals.plos.org/plosone/s/submission-guidelines#loc-laboratory-protocols

We look forward to receiving your revised manuscript.

Kind regards,

Ricardo Q. Gurgel, PhD

Academic Editor

PLOS ONE

**Response:** We appreciate your detailed reading of our manuscript and insightful comments and suggestions, as well as the opportunity to review and resubmit it. On the following sections, we have attempted to respond to all of the comments made by the editors and reviewers.

**Journal Requirements:**

**Comment1:** Please review your reference list to ensure that it is complete and correct. If you have cited papers that have been retracted, please include the rationale for doing so in the manuscript text, or remove these references and replace them with relevant current references. Any changes to the reference list should be mentioned in the rebuttal letter that accompanies your revised manuscript. If you need to cite a retracted article, indicate the article’s retracted status in the References list and also include a citation and full reference for the retraction notice.

**Response:** Thank you for recommending that we have checked the appropriateness of the references we used in the manuscript. We reviewed all of the references as per your suggestion, and none of them have retracted references.

**Comment2:** Please ensure that your manuscript meets PLOS ONE's style requirements, including those for file naming. The PLOS ONE style templates can be found at <https://journals.plos.org/plosone/s/file?id=wjVg/PLOSOne_formatting_sample_main_body.pdf> and<https://journals.plos.org/>plosone/s/file?id=ba62/PLOSOne_ formatting_sample_title_ authors_affiliations.pdf

**Response:** Thank you for your suggestion, and we do so according to the requirements of the journal.

**Commen3:** Please include additional information regarding the survey or questionnaire used in the study and ensure that you have provided sufficient details that others could replicate the analyses. For instance, if you developed a questionnaire as part of this study and it is not under a copyright more restrictive than CC-BY, please include a copy, in both the original language as well as the English version already provided, as Supporting Information.

**Response:** Thank you for your suggestion, and we have included all of the data collection tools and data sets that support the current study's findings in the supporting information section as **“S1-data collection tool for MNM”** and **“S1-Data set for MNM”** respectively.

**Comment4:** In the Methods, please clarify that participants provided oral consent. Please also state in the Methods:

- Why written consent could not be obtained

- Whether the Institutional Review Board (IRB) approved use of oral consent

- How oral consent was documented

For more information, please see our guidelines for human subjects research: https://journals.plos.org/plosone/s/submission-guidelines#loc-human-subjects-research

**Response:** Thank you for your comments, which has helped us clarify the ethical consideration section of the study. We used written informed consent, not the oral consent based on the decision of the Institutional Review Board (IRB), and we tried to make this clear in the “Ethical approval and consent to participate” section of the revised manuscript with track changes”, Page9, Line272-274

**Comment5:** You indicated that you had ethical approval for your study. In your Methods section, please ensure you have also stated whether you obtained consent from parents or guardians of the minors included in the study or whether the research ethics committee or IRB specifically waived the need for their consent.

**Response:** we appreciate your meticulous review that help us to incorporate what we have forgotten in the first draft of the manuscript. Now we have incorporated the statement regarding consent from parents or guardians of the minors and highlighted in the “Ethical approval and consent to participate” section of the “revised manuscript with track changes”, Page9, Line 275-276

**Comment6.** Please ensure you have thoroughly discussed any potential limitations of this study within the Discussion section, including the potential impact of confounding factors.

**Response:** thank you for your comment and now we have incorporated some of other limitations and we highlighted in the “strength and limitation” section of the “revised manuscript with track changes”, Page21, Line434-437.

__________________________________________________________________________________________________________END________________________________________

THANK YOU!!!

**AUTHORS’ RESPONSE TO REVIEWERS**

**DEAR: REVIEWERS**

We thank you for a thorough reading and constructive comments and suggestions of our manuscript and for the opportunity to revise and resubmit. We are pleased to submit the improved research article **“Determinants of Maternal Near Miss among Women Admitted to Maternity Wards of Tertiary Hospitals in Southern Ethiopia, 2020: A Hospital-Based Case-control Study”** for your consideration in the special collection of Plos One. On the following pages, you will find our response to the reviewers’ comments. On behalf of my co-authors, I thank you for your consideration of this resubmission. We appreciate your time and look forward to your response independently as for reviewer 1 and 2 respectively.

Sincerely,

Aklilu H. (MPH)(corresponding author)

[**aklilihabte57@gmail.com**](mailto:aklilihabte57@gmail.com)

**AUTHORS’ RESPONSE TO REVIEWER**S

1. Is the manuscript technically sound, and do the data support the conclusions?

The manuscript must describe a technically sound piece of scientific research with data that supports the conclusions. Experiments must have been conducted rigorously, with appropriate controls, replication, and sample sizes. The conclusions must be drawn appropriately based on the data presented.

Reviewer #1: Yes

Reviewer #2: Yes

______________________________________

**Response:** We thank the reviewer for kind words regarding the soundness of our study.

2. Has the statistical analysis been performed appropriately and rigorously?

Reviewer #1: Yes

Reviewer #2: Yes

________________________________________

**Response:** We thank the reviewer for kind words regarding appropriateness and rigoriousity of our work.

3. Have the authors made all data underlying the findings in their manuscript fully available?

The PLOS Data policy requires authors to make all data underlying the findings described in their manuscript fully available without restriction, with rare exception (please refer to the Data Availability Statement in the manuscript PDF file). The data should be provided as part of the manuscript or its supporting information, or deposited to a public repository. For example, in addition to summary statistics, the data points behind means, medians and variance measures should be available. If there are restrictions on publicly sharing data—e.g. participant privacy or use of data from a third party—those must be specified.

Reviewer #1: Yes

Reviewer #2: Yes

_______________________________________

**Response:** We thank the reviewer for assuring the availability of all data underlying the findings in their manuscript.

4. Is the manuscript presented in an intelligible fashion and written in standard English?

PLOS ONE does not copyedit accepted manuscripts, so the language in submitted articles must be clear, correct, and unambiguous. Any typographical or grammatical errors should be corrected at revision, so please note any specific errors here.

Reviewer #1: Yes

Reviewer #2: Yes

________________________________________

**Response:** We thank the reviewer for kind words regarding the way of presentation of the informations in the manuscript.

**AUTHORS’ RESPONSE TO REVIEWER**#**1**

**Overall information:** The authors have studied one of the most important areas in maternal health research. Studies like these are really important to assess the prevalence, trend and determinant factors of maternal near-misses across different setting. Methodologically the study seems somehow good and the conclusions were almost valid on the bases of the data presented. The data were presented clearly and appropriately. However, I can suggest some major and minor revision on the paper.

**Response:** Thank you very much for taking the time to review our work and for your positive feedback. We received Your thoughtful review, along with helpful feedback and suggestions, as a valuable contribution to our ongoing work. We've attempted to respond to your comments and suggestions in the following manner.

**Authors’ Response to Major issues raised by reviewer#1**

**Comment1:** Under introduction section it was stated that, in the current study the authors have used all the three WHO criteria to make the evaluation of maternal near miss more stringent. However under data collection tool, procedure and personnel, the authors stated that they have used WHO disease-specific criteria. [Page 2, line 19-21 and page 6, line 4]

**Response:** Thank you for your thorough review of our work and for pointing out the inconsistencies in the "data collection tool, procedure, and personnel" section of the manuscript. We'd like to let you know that our case assessment was based on the WHO's near-miss criteria which are composed of; potentially life-threatening conditions, clinical interventions, and organ dysfunctions as reported in the “Result” section of the manuscript page13, Table3. Now that we've verified that we implemented the WHO near-miss criteria, and we've highlighted it in the "data collection tool, procedure, and personnel" section of the "revised manuscript with track changes," Page6, Line182-183.

**Comment2:** Cases were selected during pregnancy, delivery or postpartum period while controls were selected during pregnancy or post-partum period. The authors did not selected controls during delivery. Hence, controls were not comparable and selection bias is likely. [Page 5, Line 2-4 and 10-12]

**Response:** Thank you for your meticulous review, and please accept our apologies for the inconsistencies encountered when writing the manuscript. We want to make sure that our cases and controls come from the same population who visited the maternity ward during pregnancy, childbirth, and the postpartum period. The statement was rewritten and highlighted in the “The population of the study” section of the "revised manuscript with track changes," Page5, Line142-145.

**Comment4:** The authors did not explain how the wealth index was assessed and analyzed. However, it has been reported under the result part. [Page 9, Table 1]

**Response:** Thank you for your comment and suggestion. We have incorporated the entire process behind the assessment of the wealth index and highlighted it in the "Data analysis" section of the "revised manuscript with track changes," Page7&8, Line211-228.

**Authors’ Response to Minor issues raised by reviewer#1**

**Comment1:** The authors indicated that they have used 1:3 ratios for cases and controls. However, the final calculated sample was not in exactly 1:3 ratio. [Page 5, line 21 and 26]

**Response:** We appreciate your detailed review of our work and accept your scientific and appropriate comment. Of course, we used a case-to-control ratio of 1:3 when calculating sample size, and the final sample size was 292(73 cases and 219 controls). However, when 10% non-response for cases and controls is taken into account, there is a small difference, which has occurred in the majority of previous studies. As a result, we kindly inform you that this minor difference was caused by the addition of 10% NR.

**Comment2**: The authors considered different hospital; this was good to avoid factors that could be unique to a given hospital as a result of the referral pattern. However, the authors did not indicate what the selected hospitals were. What the pattern of cases and controls from each hospital looks like. [Page 5, line 28].

**Response:** Thank you for your thorough review, which aided us in clearly indicating the sampling procedures. Now, we have tried to include those selected hospitals with the respective number of selected cases and controls in the "sampling procedure" section of the "revised manuscript with track changes" Page6, Line171-175,

**Comment3:** The authors stated that controls were picked using systematic methods of sampling. This needs to be elaborated on clearly. [Page5, line 32]

**Response:** We appreciate your comments and suggestions. We tried to clarify how we used the systematic sample technique and highlighted it in the “sampling procedure” section of “the revised manuscript with track changes” Page 6, Line 176-178.

**Comment4:** The authors need to clearly explain the source population for cases and controls

**Response:** thank you for your suggestion and we updated it as per your recommendation and the changes that we have made were highlighted in the “the population of the study” section of the revised manuscript with track changes,” Page 5, Line 130-134[for cases] and Page 5, Line 142-147[for controls].

**Comment5:**The authors stated they have used standard tools after possible adjustment to the local context. What adjustment was actually made? [Page 6, line 4]

**Response:** thank you for the question raised and we mean that the adjustment in terms of translation into the local language.

**Comment6:**Under result section I do not see the advantage of repetition of variables in table 2 and table 3

**Response:** Thank you for taking the time to read and comment on our manuscript. We also found it as redundant and we've replaced it with another table that properly represents the variables in "Maternal health service-related characteristics". The corresponding table was highlighted on Page15, Line334 of the revised manuscript with track changes.

**Comment7:**Remove figure 1 title from page 13.

**Response:** We appreciate your input and suggestions, but Plos One's submission guidelines recommend that the figure heading be included in the main manuscript and that the figure be submitted separately in the submission form.

**Commnet8:** The resolution for figure 1 need improvement.

**Response:** Thank you for your suggestion; we attempted to correct the current figure's resolution and submitted it as Fig1.tif

**Comment9**:In order to maintain the logical flow of results, I suggest the authors shall put maternal health service-related characteristics immediately before determinant of maternal near-miss

**Response:** Thank you for your suggestion, and we've rearranged it according to your suggestions.

**Comment10:** Under discussion part in the last paragraph the authors stated the result could not be inferred to the general population. Can they clearly specify and elaborate this statement?

**Response:** first we would like to appreciate your question. Generalizability issue might be One of the limitations behind facility based case control studies since therir migt be meany cases at the community level that couldn’t came to hospitals due to various reason. But this might be les likely and we removed it and highlighted it in “Strength and limitation” section of the “revised manuscript with track changes”, Page21, Line 436-438

**Commnet11:**Under conclusion section the authors have recommended, stakeholders at the zonal and regional levels need to enhance their efforts for improving access to roads and transportations facilities like ambulance services. I could not see any data from the study supporting this recommendation. The study did not assessed infrastructures and ambulance service. [Page19, line 31-32]

**Response:** We appreciate your detailed review of our work. That recommendation was made for a reason. Second delay (delay in reaching a health facility for obstetric care) was one of the variables significantly associated with MNM, which is primarily due to long distances to hospitals, geographical barriers, poor road networks, lack of transportation choices, poor communication, and costs. Furthermore, we attempted to determine modes of transportation for accessing health facilities, and we found that only 22.1% of the total respondents used ambulance service. As a result, we kindly inform you that the variable second delay was the justification behind the above recommendations. We hope that we have been able to partially answer your question.

**Response to Miscellaneous remarks**

**Commnet1:**Typing error, eg. Cases were not 221 rather 241 in tables under result section. The authors shall edit for some grammatical and punctuation issues. Besides, unnecessary bold letters, words, and numbers should be revised from the entire document especially under reference.

**Response:** Thank you for your helpful suggestions and comments on the typological and grammatical issues. We made corrections to the control number in each table. In the revised manuscript, we attempted to address all of the aforementioned editorial problems.

**Commnet2:** The authors did not acknowledged the strength and limitation of the study

**Response:** Thank you for your comment and suggestion; we have added some limitation of the study that were not mentioned in first draft of the manuscript and we highlighted them in the "Strength and Limitation" section of the "revised manuscript with track changes" Page21, Line434-437.

**Commnet3:**Reference should be improved in such a way that it sticks to the standard. Eg. reference 16 and 31 were super scripted. [Page 4, line 17]

**Response:** thank you for indepth review and now we have corrected and highlighted it in the “introduction” section the "revised manuscript with track changes" of Page4, Line114

__________________________________________________________________________________________________________END________________________________________

THANK YOU!!!

**AUTHORS’ RESPONSE TO REVIEWER**#**2**

Reviewer #2: Thank you for giving me this chance to review an interesting area in maternal health

**Response:** Thank you very much for taking the time to review our work and for your positive feedback. We received Your thoughtful review, along with helpful feedback and suggestions, as a valuable contribution to our ongoing work. We've attempted to respond to your comments and suggestions in the following manner.

**Commnet1:**The topic has been discussed many times in the Ethiopian context, what new information do you think you brought to the scientific world from your current study?

**Response:** We thank you for asking a reasonable question. Although a few studies have been conducted in Ethiopia, the majority of them did not rely on the WHO's full requirements (i.e. they only looked at potentially life-threatening conditions). Unlike previous research in Ethiopia, the current study was focused on three different criteria (potentially life-threatening conditions, provision of critical interventions, and organ dysfunction / life-threatening conditions), which may make the assessment criteria more stringent and provide a better picture of near-miss events.

**Commnet2**: The manuscript has no line numbers, which made the review and putting comments and questions quit challenging

**Response:** We would like to say sorry for forgetting the line number and making your review process more difficult. Now we give line numbers throughout the “revised manuscript with track changes” and the “revised manuscript”.

**Commnent3 (Abstract):** How would you justify this sentence in the abstract section “Ethiopia is one of the countries in sub-Saharan Africa with high maternal mortality and morbidity, but there is little evidence of risk factors for a near miss”? Because I have seen different studies conducted on the issue in Ethiopia

**Response:** thank you for your question. The intention behind that statement was the reason that we have mentioned in the response to your “commnet2”. In addition We kindly inform you that some of the published studies in issue were conducted after the we conduct this study. Finally, we shared your concern, so we replaced the statement with another concept and highlighted it in the "Abstract" section of the "revised manuscript with track changes," Page 2, Line 37-39.

**Commnet4(method):** On the study setting, would you describe the 5 selected hospitals of the study?

**Response:** thank you for your commnet and suggestion that was also raised by reviewer one. Now we include and highlighted in the “sampling procedure section” section of the “revised manuscript with track changes”, Page6,Line 166-168

**Commnet5(Result):**On subsection maternal health service-related characteristics, line 2, re-write as Regarding the place of birth

**Response:** thank you for your suggestion and we have corrected and highlighted in the “maternal health service-related characteristics”section of the the “revised manuscript with track changes”, Page13, Line 323-324.

**Comment:**Same section line 5: capitalize the letter t, The predominant reasons.....

**Resonse:** Thank you for your thorough analysis of our work; we corrected and highlighted it on Page 13, Line 327 of the "revised manuscript with track changes"

**Comment6(Discussion):**Paragraph 3 ,line 9: in the sentence..... presence of convincing clinical signals. Do you mean signs?

**Response:** Thank you for your feedback; we replaced the word "signal" with "indications" in the "Discussion" section of the "revised manuscript with track changes," Page 19, Line 394.

**Comment7:**The last paragraph on the discussion speaks about strength and limitation, so provide the appropriate heading for the paragraph

**Response:** Thank you for your suggestion; we followed it and highlighted it on Page 20, Line 426 of the "revised manuscript with track changes"

6. PLOS authors have the option to publish the peer review history of their article (what does this mean?). If published, this will include your full peer review and any attached files.

If you choose “no”, your identity will remain anonymous but your review may still be made public.

Do you want your identity to be public for this peer review? For information about this choice, including consent withdrawal, please see our Privacy Policy.

Reviewer #1: No

Reviewer #2: No

Response: no

Comment: While revising your submission, please upload your figure files to the Preflight Analysis and Conversion Engine (PACE) digital diagnostic tool, https://pacev2.apexcovantage.com/. PACE helps ensure that figures meet PLOS requirements. To use PACE, you must first register as a user. Registration is free. Then, login and navigate to the UPLOAD tab, where you will find detailed instructions on how to use the tool. If you encounter any issues or have any questions when using PACE, please email PLOS at figures@plos.org. Please note that Supporting Information files do not need this step.

Response: The figure was generated in accordance with the journal's guidelines.

THANK YOU!!!
