# Supplementary material for: Determinants of maternal near miss among women admitted to maternity wards of tertiary hospitals in Southern Ethiopia, 2020: A hospital-based case-control study
Source: PLoS One. 2021 May 17;16(5):e0251826. doi: 10.1371/journal.pone.0251826 (PMC8128231; doi:10.1371/journal.pone.0251826)
Supplement: Attachment — Submitted filename: Response to Reviewers.docx [file pone.0251826.s004.docx]

**AUTHORS’ RESPONSE TO ACADEMIC EDITOR AND REVIEWERS**

1. **AUTHOR’S RESPONSE TO EDITOR**

**Dear: Ricardo Q. Gurgel, PhD(Academic Editor )**

We thank you for a thorough reading and constructive comments and suggestions of our manuscript and for the opportunity to revise and resubmit. We are pleased to submit the revised research article “**Determinants of Maternal Near Miss among Women Admitted to Maternity Wards of Tertiary Hospitals in Southern Ethiopia, 2020: A Hospital-Based Case-control Study**” for your consideration in the special collection of Plos One. On the following pages, you will find our response to the editor’s comments. On behalf of my co-authors, I thank you for your consideration of this resubmission. We appreciate your time and look forward to your response.

Sincerely,

Aklilu Habte(MPH)(corresponding author)

[**aklilihabte57@gmail.com**](mailto:aklilihabte57@gmail.com)

PONE-D-20-40965R1

Determinants of Maternal Near Miss among Women Admitted to Maternity Wards of Tertiary Hospitals in Southern Ethiopia, 2020: A Hospital-Based Case-control Study

PLOS ONE

Dear Dr. Hailegebireal,

Thank you for submitting your manuscript to PLOS ONE. After careful consideration, we feel that it has merit but does not fully meet PLOS ONE’s publication criteria as it currently stands. Therefore, we invite you to submit a revised version of the manuscript that addresses the points raised during the review process.

Please make the few remainning changes for the final acceptance.

Please submit your revised manuscript by Jun 10 2021 11:59PM. If you will need more time than this to complete your revisions, please reply to this message or contact the journal office at plosone@plos.org. When you're ready to submit your revision, log on to https://www.editorialmanager.com/pone/ and select the 'Submissions Needing Revision' folder to locate your manuscript file.

Please include the following items when submitting your revised manuscript:

•A rebuttal letter that responds to each point raised by the academic editor and reviewer(s). You should upload this letter as a separate file labeled 'Response to Reviewers'.

•A marked-up copy of your manuscript that highlights changes made to the original version. You should upload this as a separate file labeled 'Revised Manuscript with Track Changes'.

•An unmarked version of your revised paper without tracked changes. You should upload this as a separate file labeled 'Manuscript'.

If you would like to make changes to your financial disclosure, please include your updated statement in your cover letter. Guidelines for resubmitting your figure files are available below the reviewer comments at the end of this letter.

If applicable, we recommend that you deposit your laboratory protocols in protocols.io to enhance the reproducibility of your results. Protocols.io assigns your protocol its own identifier (DOI) so that it can be cited independently in the future. For instructions see: http://journals.plos.org/plosone/s/submission-guidelines#loc-laboratory-protocols. Additionally, PLOS ONE offers an option for publishing peer-reviewed Lab Protocol articles, which describe protocols hosted on protocols.io. Read more information on sharing protocols at https://plos.org/protocols?utm_medium=editorial-email&utm_source=authorletters&utm_campaign=protocols.

We look forward to receiving your revised manuscript.

Kind regards,

Ricardo Q. Gurgel, PhD

Academic Editor

PLOS ONE

**Response:** Thank you very much for all of your help during the manuscript's processing. Before submitting the revised manuscript, we attempted to address the reviewers' questions as follows:

**Journal Requirements:**

Please review your reference list to ensure that it is complete and correct. If you have cited papers that have been retracted, please include the rationale for doing so in the manuscript text, or remove these references and replace them with relevant current references. Any changes to the reference list should be mentioned in the rebuttal letter that accompanies your revised manuscript. If you need to cite a retracted article, indicate the article’s retracted status in the References list and also include a citation and full reference for the retraction notice.

**Response:** Thank you for recommending that we have checked the appropriateness of the references we used in the manuscript. We reviewed all of the references as per your suggestion, and none of them have retracted references.

**AUTHORS’ RESPONSE TO REVIEWERS**

**DEAR: REVIEWERS**

We thank you for a thorough reading and constructive comments and suggestions of our manuscript and for the opportunity to revise and resubmit. We are pleased to submit the improved research article **“Determinants of Maternal Near Miss among Women Admitted to Maternity Wards of Tertiary Hospitals in Southern Ethiopia, 2020: A Hospital-Based Case-control Study”** for your consideration in the special collection of Plos One. On the following pages, you will find our response to the reviewers’ comments. On behalf of my co-authors, I thank you for your consideration of this resubmission. We appreciate your time and look forward to your response independently as for reviewer 1 and 2 respectively.

Sincerely,

Aklilu H. (MPH)(corresponding author)

[**aklilihabte57@gmail.com**](mailto:aklilihabte57@gmail.com)

**AUTHORS’ RESPONSE TO REVIEWER**S

1. If the authors have adequately addressed your comments raised in a previous round of review and you feel that this manuscript is now acceptable for publication, you may indicate that here to bypass the “Comments to the Author” section, enter your conflict of interest statement in the “Confidential to Editor” section, and submit your "Accept" recommendation.

Reviewer #1: All comments have been addressed

Reviewer #2: (No Response)

______________________________________________________________

**Response:** thank you Reviewer #1 for your kind response and we are also happy for addressing your valuable comments and suggstions. In the following sections we have tried to address the comments and suggestions raised by Reviewer #2.

2. Is the manuscript technically sound, and do the data support the conclusions?

The manuscript must describe a technically sound piece of scientific research with data that supports the conclusions. Experiments must have been conducted rigorously, with appropriate controls, replication, and sample sizes. The conclusions must be drawn appropriately based on the data presented.

Reviewer #1: Yes

Reviewer #2: Yes

___________________________________________________________________________

**Response:** We thank the reviewer for kind words

3. Has the statistical analysis been performed appropriately and rigorously?

Reviewer #1: Yes

Reviewer #2: Yes

**Response:** we appreciate you supportive comment regarding the statistical analysis of the data

4. Have the authors made all data underlying the findings in their manuscript fully available?

The PLOS Data policy requires authors to make all data underlying the findings described in their manuscript fully available without restriction, with rare exception (please refer to the Data Availability Statement in the manuscript PDF file). The data should be provided as part of the manuscript or its supporting information, or deposited to a public repository. For example, in addition to summary statistics, the data points behind means, medians and variance measures should be available. If there are restrictions on publicly sharing data—e.g. participant privacy or use of data from a third party—those must be specified.

Reviewer #1: Yes

Reviewer #2: Yes

**Response:** We thank the reviewer for assuring the availability of all data underlying the findings in their manuscript.

5. Is the manuscript presented in an intelligible fashion and written in standard English?

PLOS ONE does not copyedit accepted manuscripts, so the language in submitted articles must be clear, correct, and unambiguous. Any typographical or grammatical errors should be corrected at revision, so please note any specific errors here.

Reviewer #1: Yes

Reviewer #2: Yes

**Response:** We thank the reviewer for kind words regarding the way of presentation of the information in the manuscript.

**AUTHORS’ RESPONSE TO REVIEWER**#**1**

Reviewer#: I would like to thank the authors for their effort to consider my comment. I have thoroughly gone through the document and I found that all my comment were properly addressed. However, I am still having a concern regarding the resolution of Figure 1.

**Response:** Thank you very much for taking the time to review our work and for your positive feedback. We are also delighted to respond to your helpful feedback and comments. In the current submission, we have attempted to make Figure 1 clearer.

__________________________________________________________________________________________________________END________________________________________

THANK YOU!!!

**AUTHORS’ RESPONSE TO REVIEWER**#**2**

Reviewer #2: Thank you for addressing my previous comments. I am very impressed with the level of effort authors have made to revise their publication. But still there are some modifications.

**Response:** Thank you for taking the time to review our work after we responded to your comments and suggestions, and for recognizing our efforts in responding to your feedback. We received your thoughtful review, along with helpful feedback and suggestions, as a valuable contribution to our ongoing work. We've attempted to respond to your comments and suggestions in the following manner.

**Comment1**: 1. On the abstract section, modifying the sentence with “As a result, assessing and identifying MNM determinants will aid in improving the capacity of the health system to reduce severe maternal morbidity and mortality” will not address the concern I have raised previously. Your justification should address the importance or uniqueness of your study, removing the statement would take you back my primary concern , which was: How would you justify this sentence in the abstract section “Ethiopia is one of the countries in sub-Saharan Africa with high maternal mortality and morbidity, but there is little evidence of risk factors for a near miss”? I have seen different studies conducted on the issue in Ethiopia

**Response:** We appreciate your thoughtful response and apologize for any inconvenience this has caused. Our intention was to strengthen the abstract section by stressing the importance of studying MNM determinants. Now we have add the initial statement with a little modification in Page2, Line 38-4 of the revised manuscript as “Ethiopia is one of the countries in sub-Saharan Africa with high maternal mortality and morbidity, but there is little evidence of risk factors for a maternal near miss by using WHO criteria”.

Although a few studies have been conducted in Ethiopia, the majority of them did not rely on the WHO's full requirements (i.e. they only looked at potentially life-threatening conditions). Unlike previous research in Ethiopia, the current study was focused on three different criteria (potentially life-threatening conditions, provision of critical interventions, and organ dysfunction / life-threatening conditions), which may make the assessment criteria more stringent and provide a better picture of near-miss events. We hope that we have at least partially addressed your concern.

**Commnet2**: The line numbers should be continuous (on the manuscript you revised), and I believe while referring the points you addressed, you should mention the lines numbers on the revised manuscript.

Response: Thank you for your comment; we would take your advice.

**Commnent3:** The issue I raised regarding the description of the five hospitals should be on the study area, in addition not only mentioning the name of the hospitals but also describing the hospitals in relation to your study objective (in the terms of number of obstetrics cases treated annually, facility related characteristics like the readiness of the facilities in managing cases of MNM....)

**Response:** Thank you for your comment and suggestion, and we have tried to incorporate and highlight the ideas posed by you in the revised manuscript's "Study setting, period, and design" section, Page 5, Line 128-30.

7. PLOS authors have the option to publish the peer review history of their article (what does this mean?). If published, this will include your full peer review and any attached files.

If you choose “no”, your identity will remain anonymous but your review may still be made public.

Do you want your identity to be public for this peer review? For information about this choice, including consent withdrawal, please see our Privacy Policy.

Reviewer #1: Yes: Firew Tiyare Tiruneh

Reviewer #2: No

**Response:** No

__________________________________________________________________________________________________________END________________________________________

THANK YOU!!!
